# Relationships Between Wood-Anatomical Features and Resistance Drilling Density in Norway Spruce and European Beech

**DOI:** 10.3389/fpls.2022.872950

**Published:** 2022-04-08

**Authors:** Domen Arnič, Luka Krajnc, Jožica Gričar, Peter Prislan

**Affiliations:** ^1^Department for Forest Technique and Economics, Slovenian Forestry Institute, Ljubljana, Slovenia; ^2^Biotechnical Faculty, University of Ljubljana, Ljubljana, Slovenia; ^3^Department of Forest Yield and Silviculture, Slovenian Forestry Institute, Ljubljana, Slovenia; ^4^Department of Forest Physiology and Genetics, Slovenian Forestry Institute, Ljubljana, Slovenia

**Keywords:** wood structure, *Fagus sylvatica*, *Picea abies*, quantitative wood anatomy, xylem anatomy, wood density, increment borer

## Abstract

Environmental conditions affect tree-ring width (TRW), wood structure, and, consequently, wood density, which is one of the main wood quality indicators. Although studies on inter- and intra-annual variability in tree-ring features or density exist, studies demonstrating a clear link between wood structure on a cellular level and its effect on wood density on a macroscopic level are rare. Norway spruce with its simple coniferous structure and European beech, a diffuse-porous angiosperm species were selected to analyze these relationships. Increment cores were collected from both species at four sites in Slovenia. In total, 24 European beech and 17 Norway spruce trees were sampled. In addition, resistance drilling measurements were performed just a few centimeters above the increment core sampling. TRW and quantitative wood anatomy measurements were performed on the collected cores. Resistance drilling density values, tree-ring (TRW, earlywood width–EWW, transition-TWW, and latewood width–LWW) and wood-anatomical features (vessel/tracheid area and diameter, cell density, relative conductive area, and cell wall thickness) were then averaged for the first 7 cm of measurements. We observed significant relationships between tree-ring and wood-anatomical features in both spruce and beech. In spruce, the highest correlation values were found between TRW and LWW. In beech, the highest correlations were observed between TRW and cell density. There were no significant relationships between wood-anatomical features and resistance drilling density in beech. However, in spruce, a significant negative correlation was found between resistance drilling density and tangential tracheid diameter, and a positive correlation between resistance drilling density and both TWW + LWW and LWW. Our findings suggest that resistance drilling measurements can be used to evaluate differences in density within and between species, but they should be improved in resolution to be able to detect changes in wood anatomy.

## Introduction

European beech (*Fagus sylvatica* L.) and Norway spruce (*Picea abies* (L.) H. Karst.) are ecologically and economically important species of European forests. Beech is considered to be a plastic species that can adapt to different environmental conditions (Lindner et al., 2010; Vitasse et al., 2010; Martinez del Castillo et al., 2018). Similarly, Norway spruce is a species with high adaptive potential (Gričar et al., 2015), but it is shown to be particularly vulnerable to anticipated climate change (Bošel'a et al., 2014). Due to their economic importance, climate-growth relationships at inter- and intra-annual levels have been extensively studied in both species (Čermák et al., 2019; Muffler et al., 2020; Arnič et al., 2021; Jevšenak et al., 2021).

Previous studies have shown that, in addition to tree-ring width (TRW), wood structure is related to environmental conditions (Hajek et al., 2016; Diaconu et al., 2016b; Castagneri et al., 2017). In coniferous spruce, tracheids make up most of the xylem tissue. Earlywood is formed in spring and is characterized by tracheids with large lumen and thin cell walls, while in latewood, they have small lumen and thick cell walls (Björklund et al., 2017). Extreme weather events, such as droughts, can trigger the formation of intra-annual density fluctuations (IADFs), i.e., when latewood-like cells are formed in earlywood or vice versa (e.g., Battipaglia et al., 2016; Klisz et al., 2019). In general, beech is a diffuse-porous angiosperm species with vessels of similar size evenly distributed across a tree ring (Bosshard, 1982). However, extreme events, for example, can affect tree-ring porosity, i.e., the distribution and size of vessels within a tree ring. Thus, it can change from diffuse-porous in normal years to nearly semi-ring porous in years with extremely dry summers (Arnič et al., 2021). In coniferous spruce, wood structural changes mainly refer to tracheid features (wall thickness and lumen size; e.g., Gao et al., 2021; Stangler et al., 2021), the ratio of earlywood to latewood (Samusevich et al., 2020), and the occurrence of IADFs (Mayer et al., 2020). In beech, the changes refer to the ratio between different cell types (i.e., vessels, fibers, and parenchyma; Zheng et al., 2019) and vessel features (i.e., size, distribution, and grouping; Diaconu et al., 2016a).

In general, any changes in wood structure have been shown to influence wood properties (Chave et al., 2009; Eder et al., 2009). Wood density is considered to be one of the most important predictors of wood quality due to its correlation with the calorific value and with mechanical properties, such as hardness, stiffness, and strength (Preston et al., 2006; Chave et al., 2009; Nabais et al., 2018). In addition, the density and porosity of wood have a significant influence on adsorption and impregnability and thermal conductivity (Lesar and Humar, 2011; Plötze and Niemz, 2011). Several studies have been already conducted to evaluate how density is affected by changes in wood structure and anatomy (Peters et al., 2020). In conifers, wood density is related to tracheid/lumen size and the amount of cell wall material (Björklund et al., 2017). It is known that, due to the fixed latewood width (LWW) in spruce, wood density decreases with increasing TRW (Bouriaud et al., 2015). In diffuse-porous beech, the relationship between TRW and wood density is not significant (Bouriaud et al., 2004; Skomarkova et al., 2006; van der Maaten et al., 2012). As shown by Peters et al. (2020), wood density in beech is related to lumen area and the cell wall thickness of vessels, fibers, and axial and ray parenchyma cells. Most of these studies were conducted at a tree-ring level, evaluating variation in wood anatomy and wood density within and between tree-ring increments. However, little is known how such changes affect properties (i.e., density) of wood as an engineering material (Ross, 2010).

Wood density can vary considerably within a tree stem (Rathgeber, 2017), i.e., between earlywood and latewood in coniferous and ring-porous species (Björklund et al., 2017), between heartwood and sapwood (Fromm et al., 2001), and along tree height (Swenson and Enquist, 2008). Fast and reliable determination of wood density on standing trees is crucial for spatial- and large-scale analyses/estimates of carbon storage (Baker et al., 2004) and wood quality (Gao et al., 2017). Bulk wood density is traditionally determined volumetrically on large wood samples from felled trees, which is destructive and time consuming (Björklund et al., 2021). Similar as quantitative wood anatomy intra- and inter-annual variability in density can also be evaluated on increment cores, collected from standing trees (Bergsten et al., 2001; Chave et al., 2006, 2009; Klisz et al., 2016). An alternative way of measuring wood density in standing trees is the use of resistance drilling, whereby a handheld device is used to measure resistance to drilling with a specially shaped needle. This technology is more frequently applied in arboriculture, where it is used for assessing the presence of decay in trees. It creates less damage to the tree stem than extracting increment cores, since needle heads are usually around three millimeters wide (Fundova et al., 2018). With the use of special drilling needles that have been calibrated for assessing absolute wood density, such devices can also be used for an accurate estimation of wood density in the radial direction (Gao et al., 2017; Fundova et al., 2018). The use of such devices has the advantages of being quicker than other methods, which makes it easier to measure a larger number of trees and to capture more potential variation in wood density between trees. The main disadvantage of this method is that it nevertheless only provides an estimation of wood density. While accurate in general, it is also influenced by various factors, which need to be accounted for in the analysis. For example, drilling in larger trees increases the amount of friction compared to smaller trees and because of friction there is a linear trend in the measurements (Fundova et al., 2018). Moisture content also influences the measurements to some degree (Sharapov et al., 2020), although this trend is stronger at lower moisture contents and is probably negligible in living trees that have a moisture content above the fiber saturation point.

Although there have been studies on inter- and intra-annual variability in TRW and wood-anatomical features or density, analyses investigating relationships between wood structure on a cellular level and its effect on wood density at a macroscopic level are rare. The results can help to understand the effects of variable wood structure/anatomy on the suitability of wood as a raw or engineering material. The aim of this study was thus (i) to investigate the relationships between the selected wood-anatomical features (TRW and features of conducting cells) and resistance drilling density in the youngest seven centimeters of the stem increment in Norway spruce and European beech and (ii) to evaluate the potential of resistance drilling measurements for studies on the effect of variations in wood anatomy on wood density. We hypothesized that (i) the relationships between TRW and features of conducting cells exist in both species at a tree ring as well as macroscopic level; (ii) the relationships between the selected anatomical features and resistance drilling density differ between coniferous spruce and diffuse-porous beech wood.

## Materials and Methods

### Site, Species, Tree Selection, and Sampling

**Figure 1:**
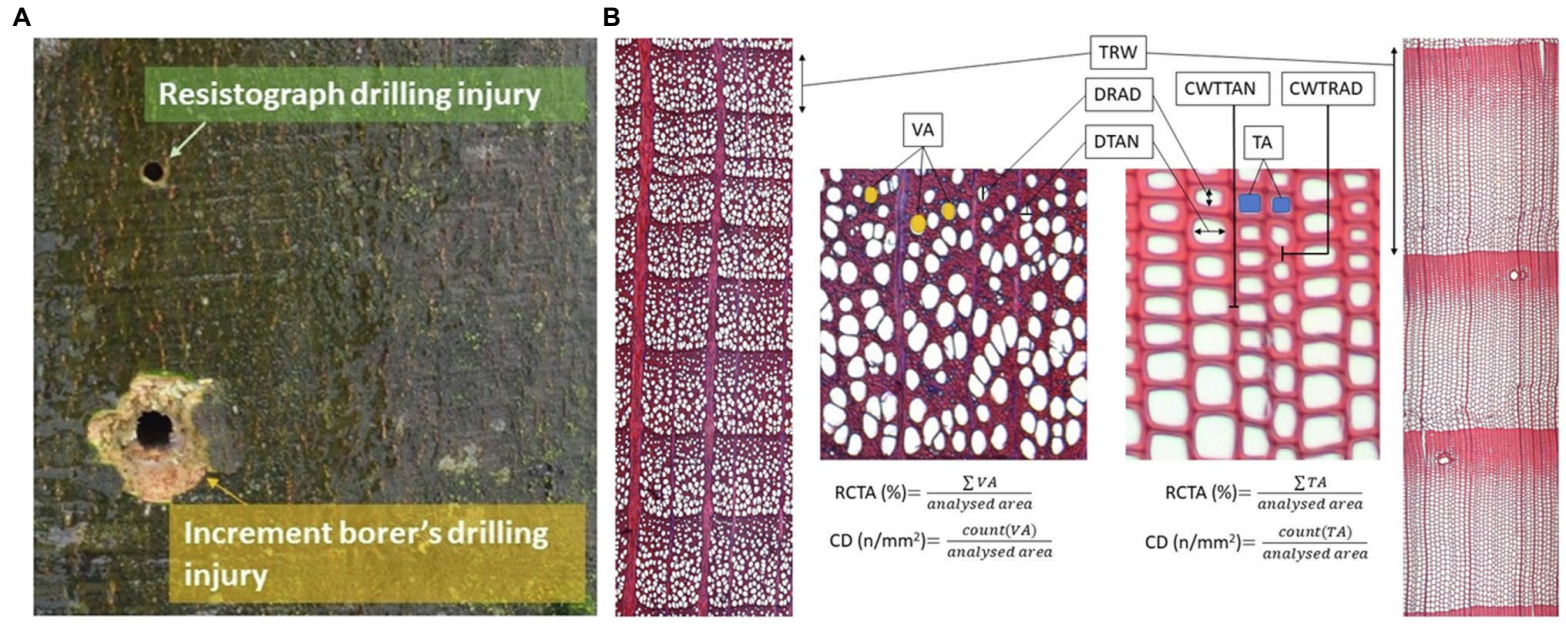
Sampling and analysis of quantitative wood anatomy. **(A)** Increment cores were collected with a 5 mm increment borer while the resistance measurements were performed with a Resistograph R650-SC 3–4 cm above the increment borer sampling point. **(B)** Measurements of tree-ring widths (TRW) and wood-anatomical features (vessel area—VA, tracheid area—TA, radial and tangential diameter of vessels and tracheids—DRAD and DTAN, radial tracheid wall thickness—CWTRAD, and tangential tracheid wall thickness—CWTTAN in spruce).

Sampling and measurements of European beech (*Fagus sylvatica* L.) and Norway spruce (*Picea abies* Karst.) were carried out at four sites in Slovenia to evaluate the relationship between wood-anatomical features and resistance drilling density. The sampling sites are representing Slovenian mountain forest with average altitude 1,155 ± 160 m above sea level, mean temperature of 7.2 ± 1.5°C, and 1,410 ± 150 mm of annual sum of precipitations in last 70 years (Supplementary Table 1; Supplementary Figure 1; Cornes et al., 2018). On those sites, a total of 24 European beech and 17 Norway spruce old grown trees (DBH > 45 cm, tree height > 29 m) were sampled and measured in a natural stand between autumn 2019 and spring 2020. Two cores bark-to-pith were extracted at breast height from different sides of each individual tree using a 5-mm increment borer (Haglöf Sweden, Långsele, Sweden). Resistance drilling density measurements were taken 3–4 cm above the increment borer sampling point using a Resistograph R650-SC (Rinntech, Heidelberg, Germany) resistance borer (Figure 1A). Based on the diameter at breast height (DBH) of the tree, drilling was done to the approximate pith of each tree (i.e., half of the DBH, similarly to increment cores).

### Measurements of Tree-Ring Widths and Wood-Anatomical Features

Measurements of wood-anatomical features and tree-ring width (TRW) were performed on 24 selected cores for beech and 17 cores for spruce. Each wooden core was split into segments of length from 3 to 4 cm. In the case of beech, a 20 μm thick transverse section was cut from each segment with a WSL sledge microtome using OLFA-80 × 9 mm spare blades (Gärtner et al., 2015). The Norway spruce segments were dehydrated in a graded series of ethanol and infiltrated with UltraClear (Avantor Performance Materials, Deventer, Netherlands) and paraffin (Paraplast plus, Leica Biosystems, Richmond, United States). After the infiltration of wood tissue, samples were embedded in paraffin blocks to stabilize the samples for further processing (Prislan et al., 2013). Transverse sections of 20 μm thickness were cut with a Leica RM 2245 rotary microtome (Leica Microsystems, Wetzlar, Germany) using Leica 819 Low Profile Microtome blades (Leica Biosystems, Nussloch, Germany). The sections were transferred to object glasses and the paraffin was then washed out with UltraClear and ethanol. The beech sections were treated with bleaching solution (5–15% chlorine content) to improve the staining intensity. The sections of both species were stained with a water mixture of safranin and Astra-blue and finally, the permanent slides were prepared using Euparal mounting medium (Arnič et al., 2021; Prislan et al., 2022).

To obtain macro-images of samples, the permanent slides were firstly scanned with a 4800×4800 dpi Color Image scanner (Epson Perfection V19, Seiko Epson Corporation, Japan). The captured figures served for the measurements of TRW using CooRecorder & CDendro software (Cybis, Saltsjöbaden, Sweden). The final cross-dating was done using PAST-5 (SCIEM, Brunn, Austria) software. TRW measurements of individual trees served as control for accurate tree-ring dating in subsequent wood anatomy analyses.

To perform quantitative wood-anatomical measurements, high-resolution images (beech: 0.514 pixel/μm, spruce: 2.056 pixel/μm) of the sections were prepared using a Leica DM 4000 B light microscope (Leica Microsystems, Wetzlar, Germany) at 50× (for beech) and 100x (for spruce) magnification with a Leica DFC 280 digital camera (Leica Microsystems, Wetzlar, Germany) and LAS image analysis software (Leica Application Suite, Leica Microsystems, Wetzlar, Germany). Image-sequences of the xylem rings were captured with at least 25% of the overlapping area and then merged using PTGui v11.16 Pro (New House Internet Services B.V., Rotterdam, Netherlands). Panoramic figures of both species were then processed with the image analysis software Image-Pro Plus 7.1 (Media cybernetics, Rockville, United States) and ROXAS (v3.0.437) to obtain the wood-anatomical features (von Arx and Carrer, 2014; von Arx et al., 2016).

**Figure 2:**
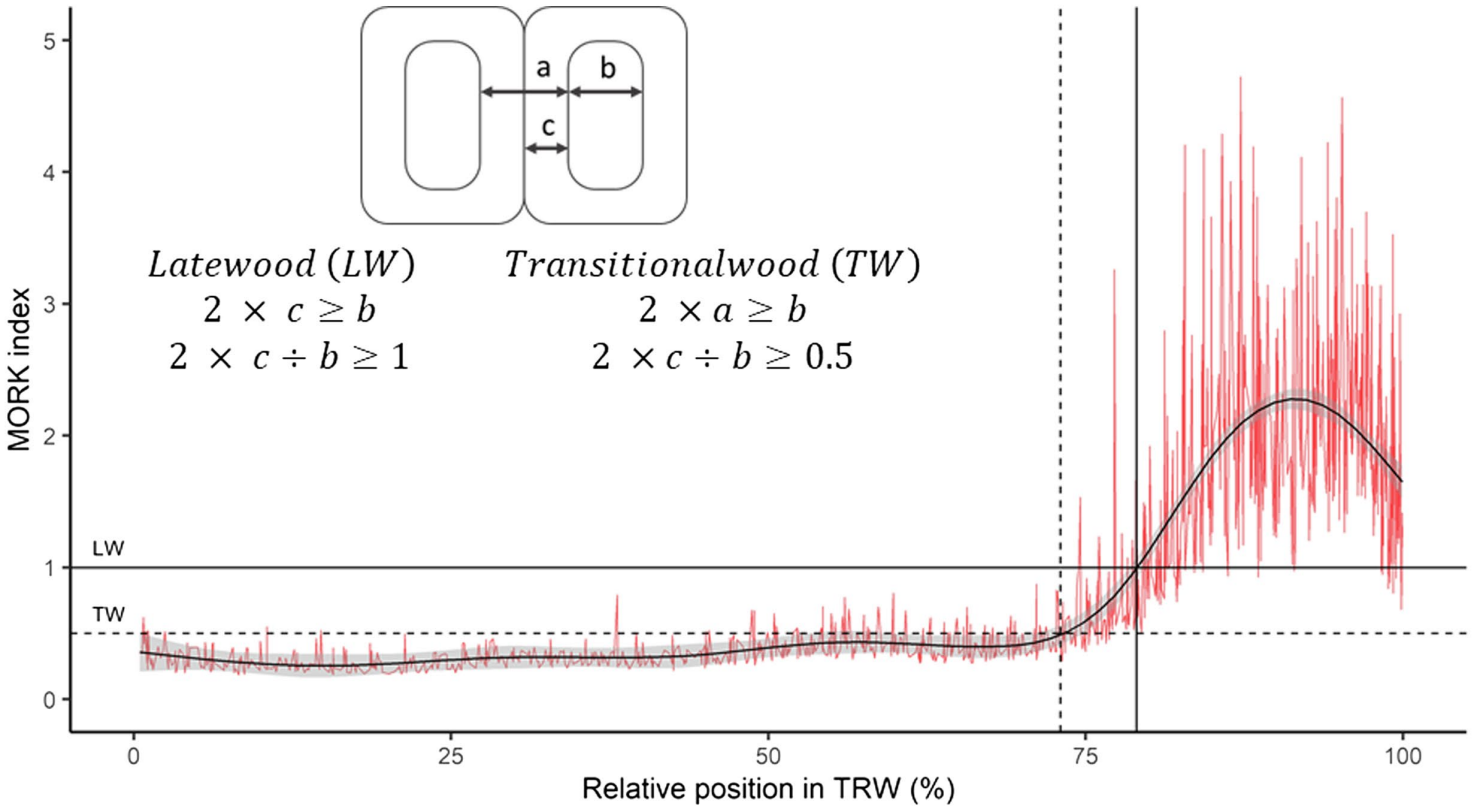
Example of latewood and transitional wood determination within spruce tree ring. The red line shows the relationship between the double thickness of the radial tracheid wall and the radial diameter of the lumen, while the black curve represents a smoothing function (GAM).

Wood-anatomical measurements were performed for both species for tree rings formed between 1960 and 2019. In each ring, the following wood-anatomical features were measured (1) mean vessel lumen area (MVA) in beech and tracheid lumen area (MTA) in spruce, (2) cell density (CD) as the number of conduit cells per square mm, (3) relative conductive area (RCTA) representing the percentage of cumulative conductive area within the measured area, (4) mean radial cell diameter (DRAD) measured in a bark-to-pith direction, and (5) mean tangential cell diameter (DTAN; measured tangentially to pith). Radial (CWTRAD), tangential (CWTTAN), and mean cell wall thickness (CWTALL) were additionally measured in spruce (Figure 1B). Based on radial tracheid cell wall thickness and radial lumen diameter, we evaluated the distance of the latewood and transitional wood by the first and second Mork’s interpretations (Denne, 1989; Figure 2). In further analyses, latewood width (LWW) and sum of latewood and transitional wood width (TWW + LWW), as well as their shares in TRW (share of latewood—LWS, the sum of shares of latewood and transitional wood—TWS + LWS), were used.

### Resistance Drilling Density Data Analysis

**Figure 3:**
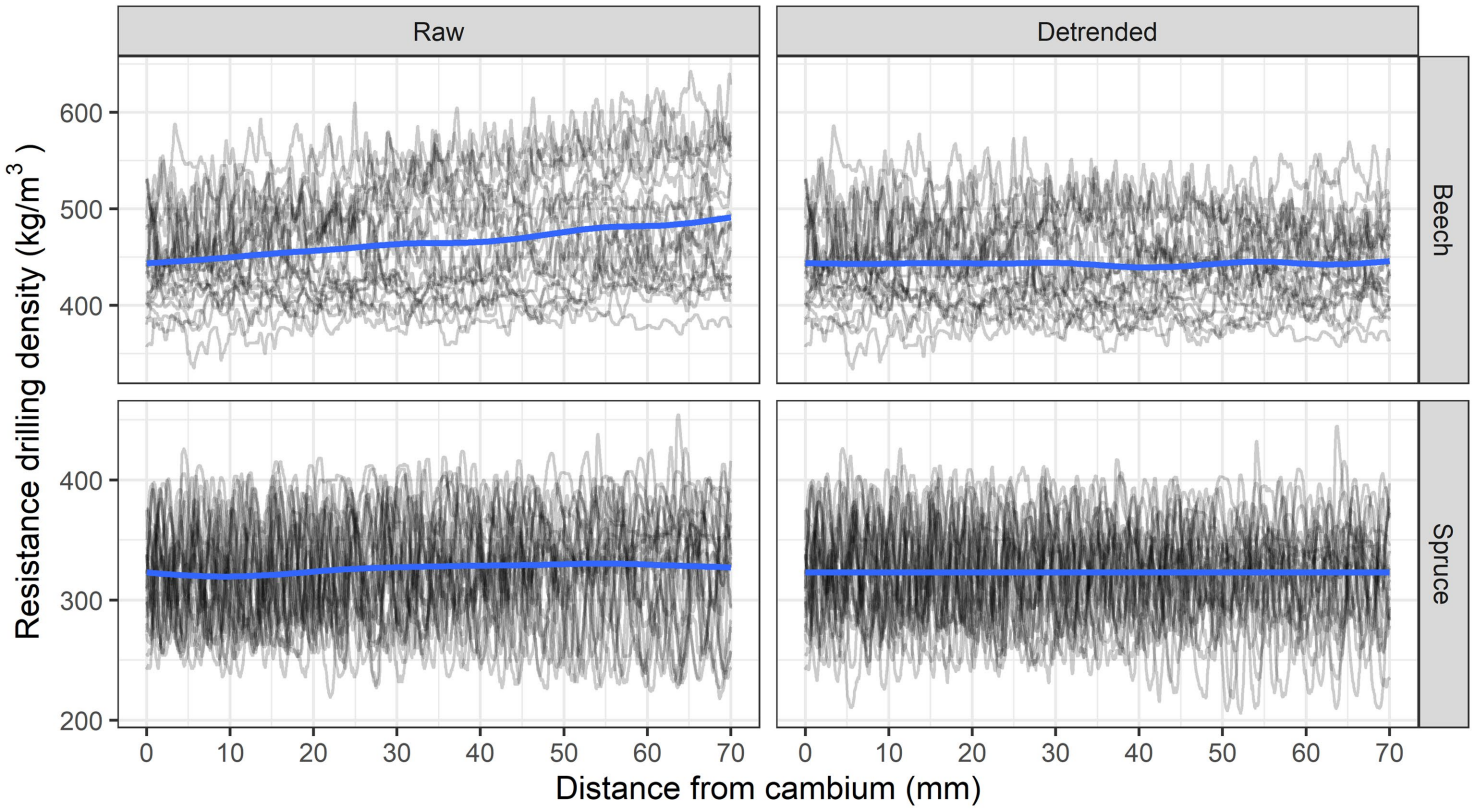
Unadjusted (raw) and detrended debarked resistance drilling density profiles for beech and spruce.

The resistance drilling measurements were imported into the R statistical environment using the R (R Core Team, 2021) package *densitr*, which enables further manipulation of the measurements in R (Krajnc et al., 2021). Wood density profiles of each tree were first trimmed to exclude the bark and determine the point of approximate cambium. Since the wood anatomy analyses were performed for the period between 1960 and 2019 and because some trees had been growing slowly during this period, we decided to standardize the analyses for each tree to the first 7 cm of wood (in the direction from cambium to pith). The wood density profiles were thus secondarily trimmed at a distance of 7 cm from the approximate cambium location and detrended by removing the linear trend due to friction using the functions from the *densitr* R package (Figure 3).

### Wood-Anatomical Features and Wood Density Relationship Analyses

Wood-anatomical and density analyses were performed in open-source statistical environment R. Since low annual wood density variability and narrow TRW were found within the majority of trees, it was not possible to determine TRW within resistance drilling density profiles. We were thus unable to perform the analyses on an annual scale and therefore decided to calculate mean values of each wood-anatomical feature and resistance drilling density for the whole 7 cm for each analyzed tree (Supplementary Figure 2).

Since the Roxas output data for wood-anatomical variables (MVA and MTA, DRAD, DTAN, and RCTA) are given on annual scale, all wood-anatomical features were weighted by TRW to calculate weighted mean anatomical variables for the whole 7 cm of wood samples. Additionally, for a better insight into resistance drilling density and into wood-anatomical variability within species, we calculated the 5th-, 10th-, 25th-,75th-, 90th-, and 95th-quantiles of resistance drilling density and selected wood-anatomical features for both species (TA and CWTALL for spruce, and VA for beech).

To determine the most pronounced relationships, Pearson correlation coefficients were calculated between wood-anatomical features and wood density quantiles. Furthermore, simple linear regression models were performed between selected resistance drilling density quantiles (mean resistance drilling density for both species and additionally 90th quantile of resistance drilling density for spruce) and selected wood-anatomical features (mean size of conduit cells—MVA and MTA, DRAD, DTAN, RCTA, and TRW for both species, and additional CWTALL, LWW, and LWW + TWW for spruce). Detailed information about used linear regression models are listed in Supplementary Tables 4–6.

## Results

### Wood-Anatomical Features in Spruce and Beech

**Figure 4:**
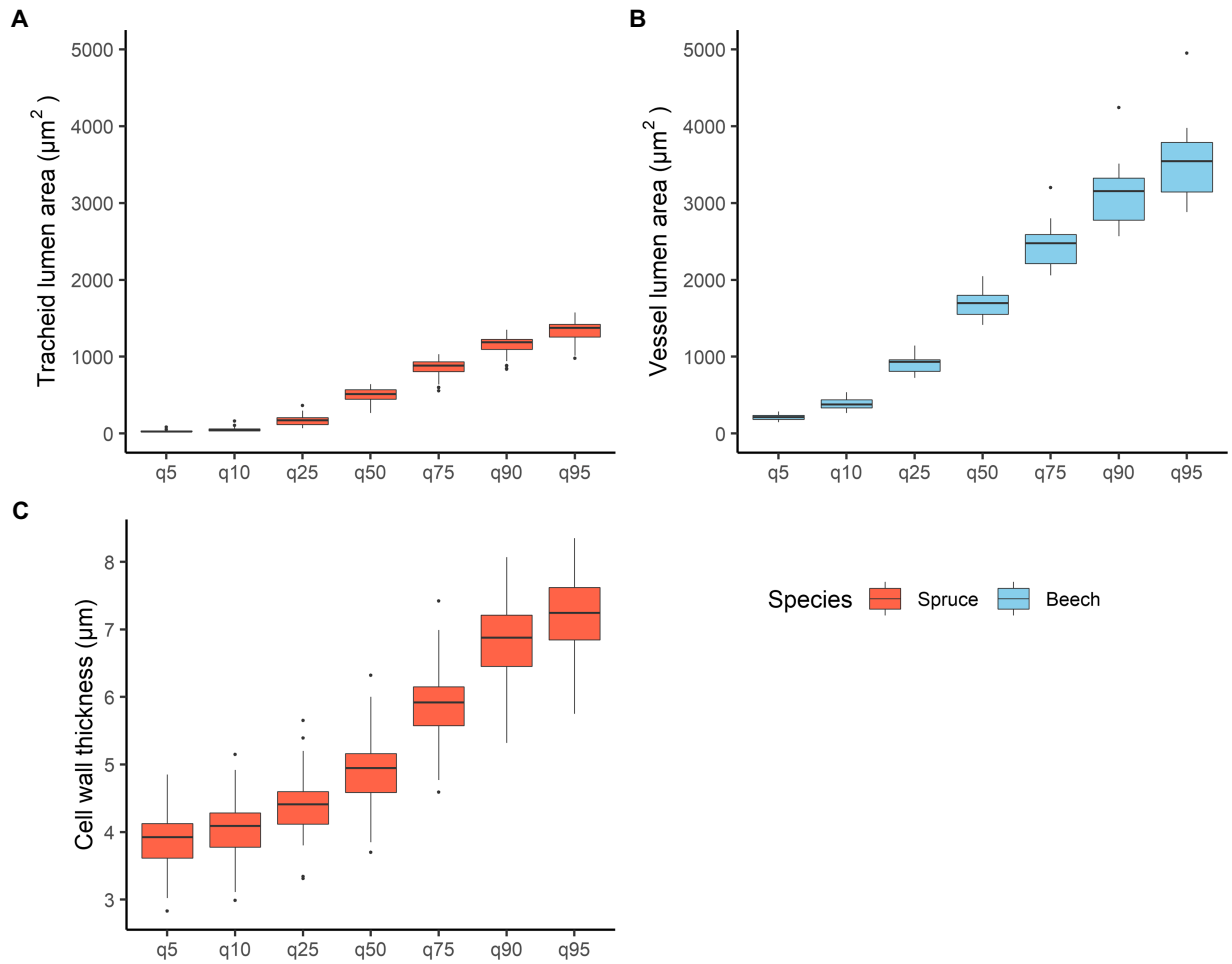
Variation in **(A)** mean spruce tracheid lumen area, **(B)** mean beech vessel lumen area, and **(C)** mean tracheid cell wall thickness within the 5th, 10th, 25th, 75th, 90th, and 95th quartiles. Quantiles were calculated on 7 cm wood samples for both species.

In spruce, TRW was 1.78 ± 0.6 mm (mean ± standard deviation), earlywood width (EWW) 1.5 ± 0.5 mm, and latewood (LWW) 0.27 ± 0.17 mm. The total width of transition wood and latewood (TWW + LWW) was 0.68 ± 0.37 mm. Minimum (5th quantile) and maximum (95th quantile) tracheid lumen area was 30.5 ± 14.1 μm^2^ and 1,316 ± 170 μm^2^, respectively, with mean values of 552 ± 86 μm^2^ (Figure 4A). Tangential and radial lumen diameters of tracheids were 23.7 ± 1.7 μm and 25.4 ± 2.9 μm, respectively. Relative conductive area was 48.1 ± 5.3%. Mean tracheid wall thickness was 5.3 ± 0.6 μm (Figure 4C).

In spruce, a high positive correlation was found between TRW and LWW (*ρ* = 0.84), and between the width of TWW+ LWW and LWW (*ρ* = 0.96; Supplementary Table 2). Furthermore, LWW and TWW + LWW showed a negative correlation with the relative conductive area (LWW: *ρ* = −0.59, TWW + LWW: *ρ* = −0.63). Relative conductive area showed a positive correlation with mean tracheid lumen area (*ρ* = 0.85) and radial (*ρ* = 0.88) and tangential (*ρ* = 0.69) tracheid diameters, whereas the correlation with mean-, radial-, and tangential cell wall thickness was negative (*ρ* = −0.94, *ρ* = −0.93, *ρ* = −0.94). In contrast, cell density showed a negative correlation with mean tracheid lumen area (*ρ* = −0.74) and radial (*ρ* = 0.67) and tangential (*ρ* = 0.75) lumen diameters, while there was no correlation with wall thickness in any direction (Supplementary Table 2).

In beech, TRW was 1.82 ± 0.4 mm. Vessel lumen area varied from 210 ± 37 μm^2^ in the 5th quantile to 3,534 ± 458 μm^2^ in the 95th quantile (Figure 4). Tangential and radial vessel lumen diameters were 49.9 ± 2.3 μm and 40.5 ± 2.4 μm, respectively. The mean vessel area was 1750 ± 183 μm^2^, whereas the relative conductive area was 20.2 ± 3.2%. Cell density showed the highest negative correlations with TRW (*ρ* = −0.55). In addition, high positive correlations were found between mean vessel area and tangential and radial vessel diameters (*ρ* = 0.96 and *ρ* = 0.84, respectively), as well as between mean vessel area and relative conductive area (*ρ* = 0.74; Supplementary Table 3).

### Variability in Resistance Drilling Density in Spruce and Beech

**Figure 5:**
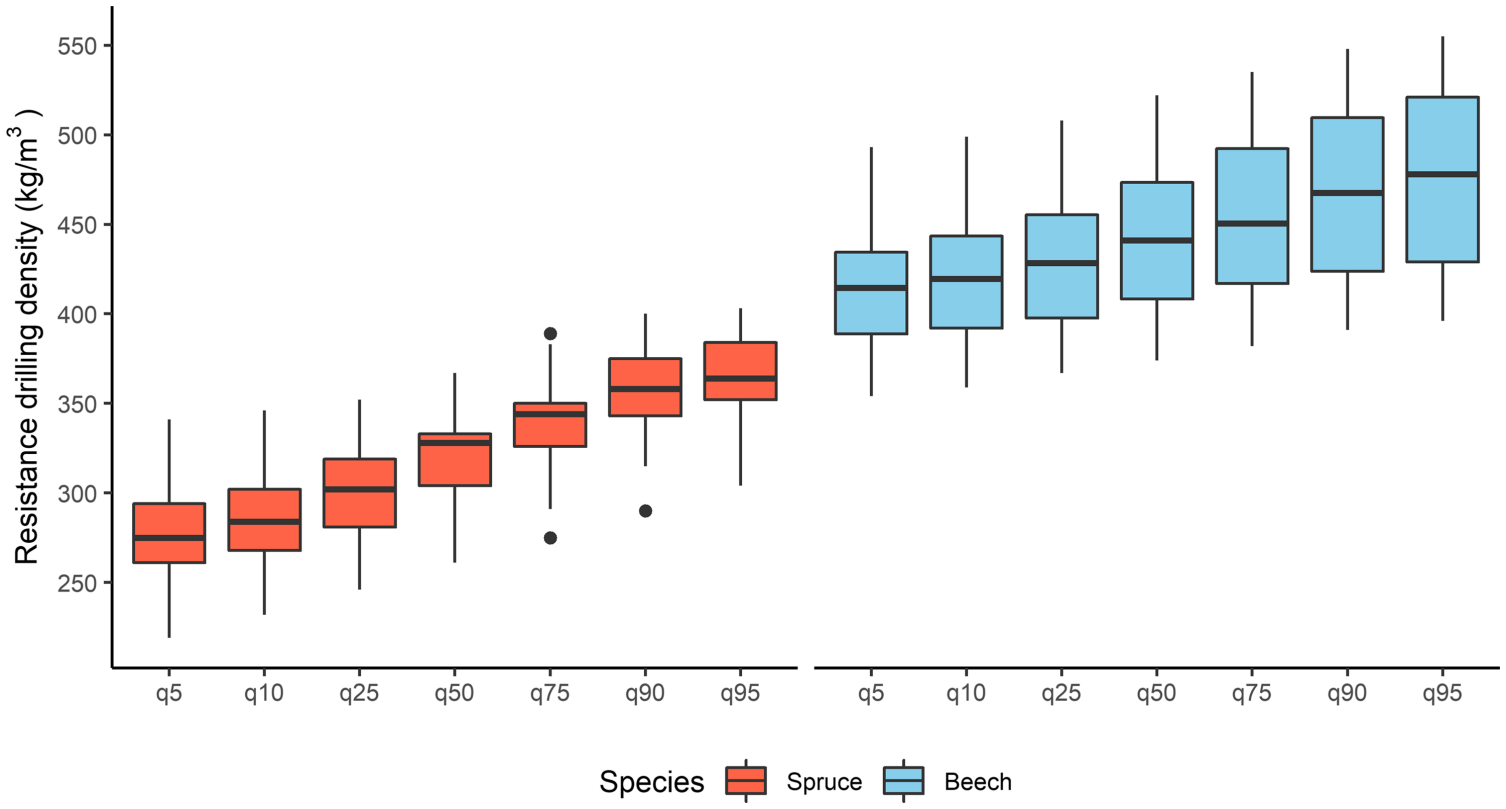
Variation in mean resistance drilling density values for spruce and beech and variation within the 5th, 10th, 25th, 75th, 90th, and 95th quartiles.

For spruce, the resistance drilling density ranged from 277 ± 31 to 365 ± 27 kg/m^3^ (mean ± standard deviation). The lowest and highest measured values ranged from 219 to 341 and 304 to 403 kg/m^3^, respectively. The mean resistance drilling density of spruce was 320 kg/m^3^ (Figure 5). In beech, resistance drilling density ranged from 414 ± 34 to 475 ± 48 kg/m^3^. The lowest and highest measured values ranged from 354 to 493 and 396 to 555 kg/m^3^, respectively. The mean resistance drilling density of beech was 441 kg/m^3^.

### Relationships Between Resistance Drilling Density and Wood-Anatomical Features

**Figure 6:**
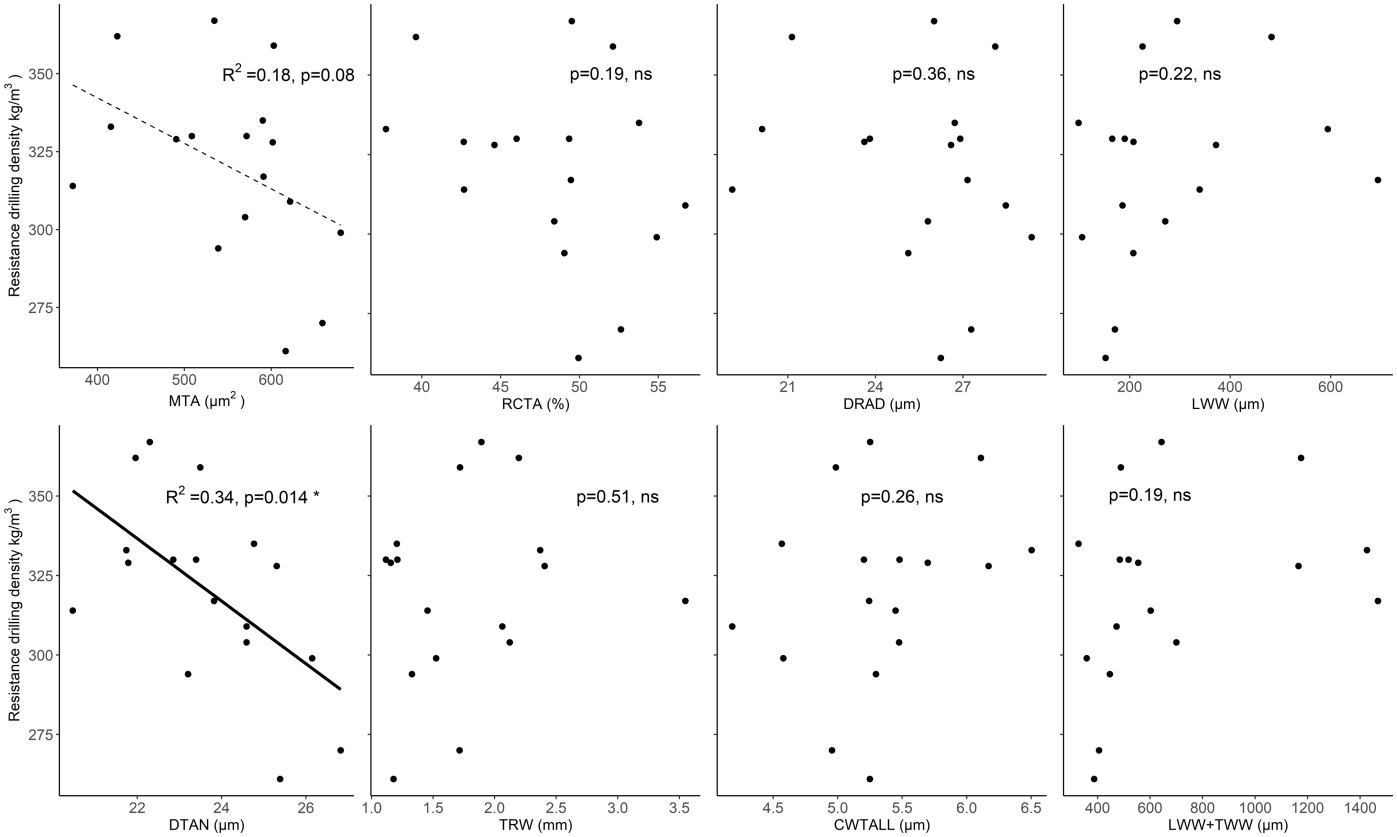
Relationships between resistance drilling density values and wood-anatomical features (MLA—mean lumen area, RCTA—relative conducting area, DRAD—radial lumen diameter, LWW—latewood width, DTAN—tangential lumen diameter, TRW—tree-ring width, CWTALL—mean tracheid wall thickness, and TWW + LWW—the total width of transition wood and latewood) in spruce. Weak relationships (*p* < 0.1) between wood-anatomical features and resistance drilling density are marked with a dotted line, while significant relationships (*p* < 0.05) are shown by a full line. Further statistical information for the presented linear models is given in Supplementary Table 4.

In spruce, mean resistance drilling density increased with increasing TRW, but this relationship was not significant. In addition, a non-significant positive relationship was found between drilling density and LWW. Most of the analyzed wood-anatomical features showed no significant relationship with drilling density. A weak negative relationship (*p* < 0.1) was found between drilling density and mean tracheid lumen area. While a significant negative relationship was found between drilling density and the tangential lumen diameter of tracheids, i.e., when tangential lumen diameter or area of tracheid’s increased, measured resistance drilling density decreased (Figure 6; Supplementary Table 4).

**Figure 7:**
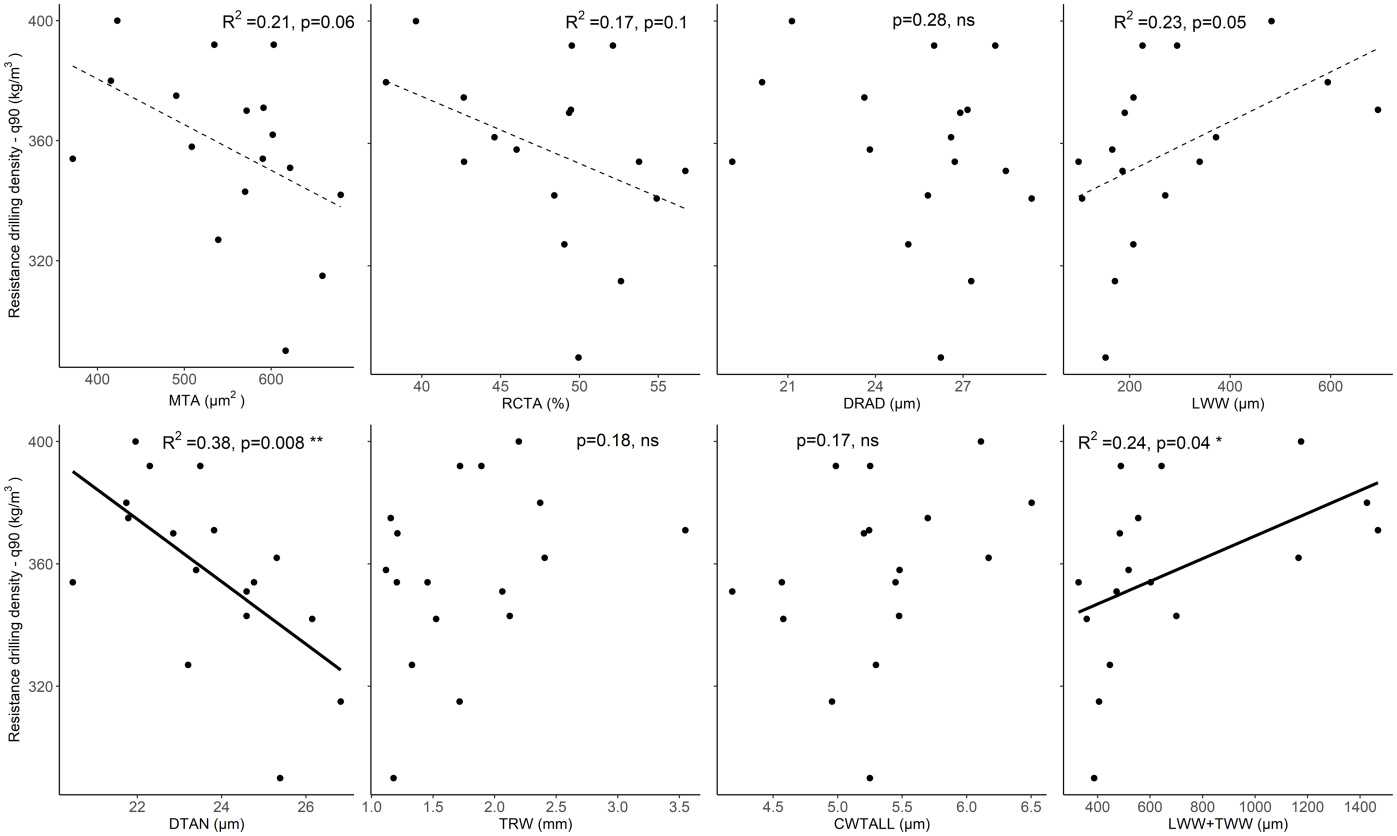
Relationships between maximum resistance drilling density (90th quantile) values and wood-anatomical features (MLA—mean lumen area, RCTA—relative conducting area, DRAD—radial lumen diameter, LWW—latewood width, DTAN—tangential lumen diameter, TRW—tree-ring width, CWTALL—cell wall thickness, and TWW + LWW—the total width of transition wood and latewood) in spruce. The weak relationship (*p* < 0.1) between wood-anatomical features and resistance drilling density is marked with a dotted line, while significant relationships (*p* < 0.05) are shown by a full line. Further statistical information for the presented linear models is given in Supplementary Table 5.

When evaluating the relationships with the nearly maximum resistance drilling density values (90th quantile), the correlations with LWW and TWW + LWW were positive and significant, while correlations with tracheid DTAN were significantly negative (Supplementary Table 2). Furthermore, weak (*p* < 0.1) and significantly negative relationships were found for mean tracheid area and relative conductive area, i.e., resistance drilling density decreased with increasing mean tracheid lumen area and relative conductive area (Figure 7; Supplementary Tables 2, 5).

**Figure 8:**
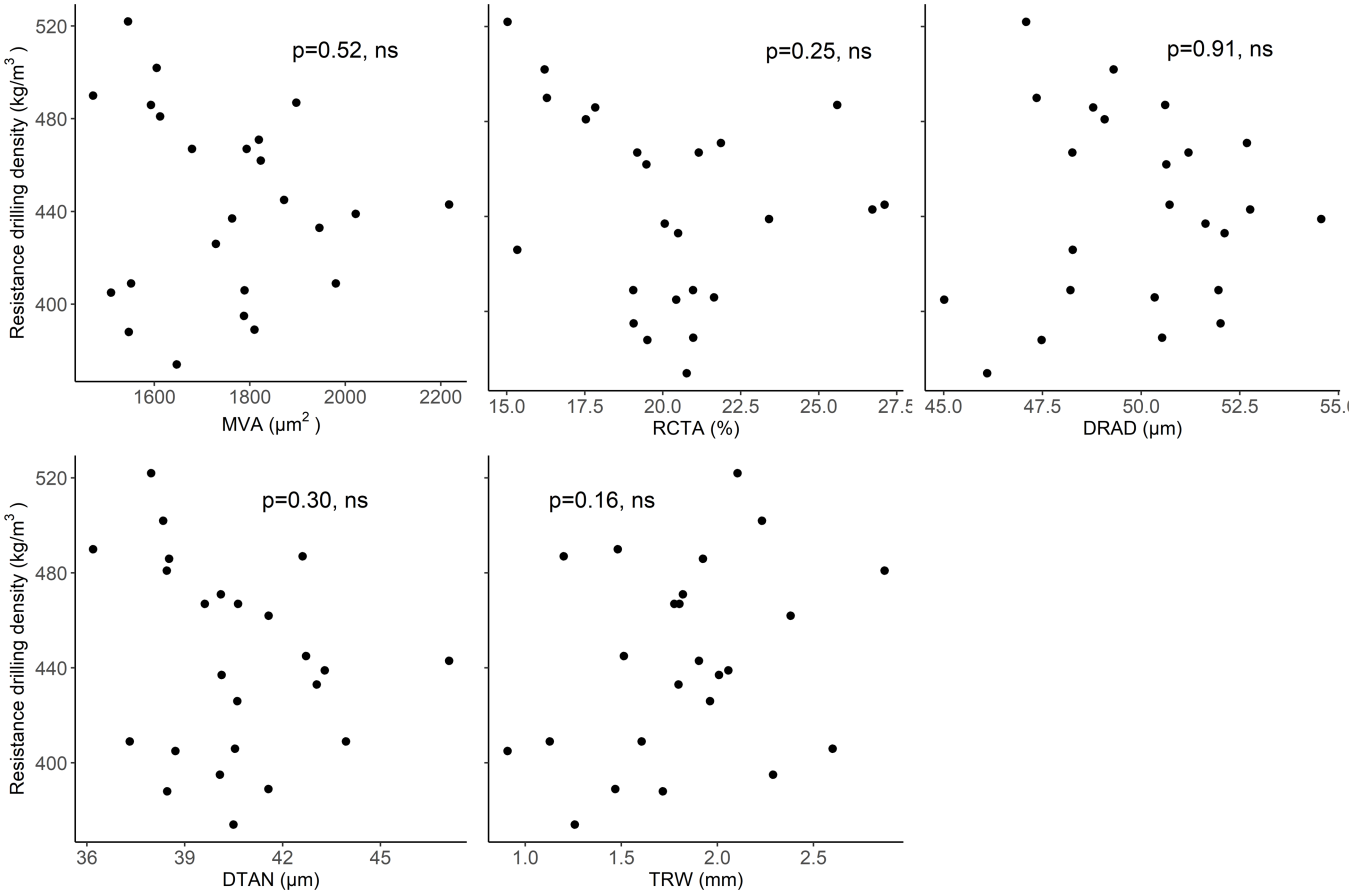
Relationships between mean resistograph values and wood-anatomical features (MLA—mean lumen area, RCTA—relative conducting area, DRAD—radial lumen diameter, DTAN—tangential lumen diameter, and TRW—tree-ring width) in beech.

In beech, there was no clear relationship between resistance drilling density and TRW. Furthermore, no relationships were found between resistance drilling density and wood-anatomical features over the examined length (Figure 8; Supplementary Table 6). The results were similar whether maximum or minimum resistance drilling density values were evaluated.

## Discussion

We confirmed our first hypothesis on the significant relationships between tree ring (i.e., TRW and ratio of EWW to LWW) and analyzed conduit features in both spruce and beech. In spruce, the highest correlation values were found between TRW and LWW. Among the analyzed anatomical features, radial cell wall thickness showed the highest correlation with TRW. Surprisingly, there was no significant relationship between tracheid lumen area and TRW. In beech, vessel density was shown to have the highest influence on TRW. Our second hypothesis was confirmed only partly because there were no significant relationships between wood-anatomical features and resistance drilling density in beech. However, for spruce, a significant negative relationship was found between resistance drilling density and tangential tracheid diameter and a positive correlation with TWW + LWW and LWW.

### Relationship Between Tree Ring, Wood-Anatomical Features, and Resistance Drilling Density

When comparing the tree-ring and wood-anatomical features in beech, significant negative relationships were found between TRW and vessel density, meaning that in wider tree rings, the number of vessels is smaller. A smaller number of vessels within a growth ring is further related to a smaller relative conductive area. These relationships were also confirmed by previous studies on beech (i.e., Oladi et al., 2014; Diaconu et al., 2016b; Arnič et al., 2021). The relationship between wood anatomy and density depends on wood porosity. This would suggest that, in wider growth rings, the share of thick-walled fibers is higher, which positively affects wood density. Peters et al. (2020) found that the density of wider tree rings in beech is higher by 0.0313 g/cm-3 per mm. In addition, they found that vessel density and vessel area are the main features affecting the density of tree rings. However, several studies have suggested that in diffuse-porous beech, the relationship between TRW and wood density is not significant (Bouriaud et al., 2004; Diaconu et al., 2016b), while others suggest that density decreases with increasing TRW (Pretzsch et al., 2018). The differences in results between studies can be explained by different methodological approaches; Peters et al. (2020), for example, calculated wood density based on quantitative wood anatomy data (measuring vessels and fibers but excluding parenchyma tissue) to estimate inter-annual variability in density, while Bouriaud et al. (2004) and Diaconu et al. (2016b) used *X*-ray densitometry. Furthermore, the differences may also be related to the high plasticity of beech, which is able to adjust vessel size and distribution (e.g., Hajek et al., 2016; Arnič et al., 2021) and cell wall thickness (e.g., Skomarkova et al., 2006; van der Maaten-Theunissen et al., 2012) depending on inter-annual variation in environmental conditions, which is reflected in the change of porosity from diffuse-porous to semi-porous (e.g., Arnič et al., 2021).

In conifers, the relationships between TRW and wood anatomy are less ambiguous, since they are mainly composed of tracheids. Increasing TRW leads to a higher proportion of earlywood with low density, which in turn leads to lower wood density. However, these relationships can change because of the frequent presence of E type of IADFs (i.e., latewood-like cells in earlywood; Krajnc et al., 2021), whose formation is under environmental (De Micco et al., 2016) as well as genetic control (Klisz et al., 2016, 2019). In case of L type IADFs (i.e., earlywood-like cells in latewood), the density may decrease even more (e.g., Battipaglia et al., 2016). Piermattei et al. (2020) analyzed several tree-ring and wood-anatomical features in spruce; they found a strong positive relationship between TRW and earlywood width but also with the radial and tangential cell wall thickness ratio. In our study, the strongest correlation was found between TRW and transitional as well as latewood width. Our results also show that relative conductive area increases with radial and tangential tracheid diameter and decreases with radial and tangential cell wall thickness. Luostarinen et al. (2017) showed that the above-mentioned parameters affect wood density in different spruce clones.

The relationships between wood structure/anatomy and density are influenced by many other factors, such as genetics and environment, which are not yet fully understood. Consequently, the relationship between earlywood and latewood is not linear with TRW and the structure of earlywood and latewood (i.e., tracheid features) is also not homogeneous and may change in response to various internal and external factors (Rao et al., 1997). Overall, all these changes affect the density and properties of the wood.

In our study, a significant negative correlation was found only between resistance drilling density and tangential tracheid diameter, as well as a positive correlation with TWW + LWW and LWW. In contrast, no correlation was found between wood-anatomical features and resistance drilling density in beech. The relationship between tangential tracheid diameter and resistance drilling density in spruce is probably a consequence of the drilling needle orientation when drilling in the radial direction. The major cutting surface of the needle cuts through the tracheids in the tangential direction, which is why a relationship was identified between those two factors. Such a relationship cannot be expected in diffuse-porous wood of beech due to the more variable wood structure, which varies in a smaller space than the minimum spatial resolution of the resistance drilling device to detect such changes.

### Differences Between Beech and Spruce in Resistance Drilling Density/Wood-Anatomical Features Correlations

The above-mentioned differences in the findings between beech and spruce can thus be explained by: (i) the differences in porosity between spruce and beech; (ii) the species-specific growth and structural response to local site conditions; and (iii) the different methodological approach.

A different methodological approach was used in this study to analyze the wood structure of spruce and beech. We focused only on analyses of the conductive cell features, i.e., the tracheids in spruce and the vessels in beech. While tracheids make up most of the wood tissue in spruce (about 95%), vessels make up 25–50% of the wood in beech (Wagenführ, 2007). Other axially oriented cells, such as fibers and axial parenchyma, account for 25–60% and 3.5–7% of wood in beech, respectively. In addition, there is also a difference between the two species in the proportion of rays, which were not included in the analyses and account for 4.4–5.5% and 11–21% of the tissue in spruce and beech, respectively (Wagenführ, 2007). These numbers show that, in the case of spruce, significantly more cells were included in the analysis (practically all of them), whereas in the case of beech, this proportion was always less than 50%, since it contained only conducting cells (i.e., vessels). The methodological approach influenced the results. We measured the relative conductive area as 50% for spruce, but 20% for beech. Because only conductive cells were included in our analyses, the values are lower than wood porosity, which is calculated from the proportions of the density of absolutely dry wood and the apparent density of cell walls (1.5 g/cm^3^; Plötze and Niemz, 2011). The calculated porosity, which includes all cell types in the wood, is 71.4% for spruce and 54.7% for beech. The low proportion of cells in beech included in the analysis undoubtedly affected the relationship between wood-anatomical features and resistance drilling density. Whether this relationship would improve in diffuse-porous beech if more cells (i.e., thick-walled fibers) were included in the analyses remains an open question for future studies.

### Potential of Resistance Drilling Measurements to Evaluate Wood Anatomy-Related Variations in Wood Density

The current study examined the possibilities of linking resistance drilling density to wood anatomy over a relatively short length of seven centimeters. Very few links were found and most of them were not very pronounced. Better relationships could perhaps be obtained if the data were examined over a fixed number of tree rings. However, this was not possible due to big differences in TRW between trees; a fixed length was therefore chosen instead. Poor relationships between wood-anatomical features and resistance drilling density are most likely related to the resolution of the resistance drilling device. Although the device used in the current study records a measurement of resistance drilling density every 1/100 of a millimeter, this measurement is an amalgamation of the measurements of two cutting surfaces of the drilling needle. The needle cuts wood along two different axes, being shaped like a triangle, with the leading spike in the middle. The practical spatial resolution of the device is therefore partly dictated by the height of the spike above the flat cutting surface (in our case, around 0.5 mm), any features smaller than that will probably not be detected accurately. This also explains why some relationships were found in spruce and none in beech, since spruce wood is more homogenous and does not vary considerably within the practical resolution size of the device. The relationships would probably improve if a different needle shape, with only one cutting surface, was used, as examined by Sharapov et al. (2020) and as mentioned by evaluating these relationships by including measurements of fiber features. The typical needle shape (triangular with a leading spike) used by default in most resistance drilling devices nowadays makes them less appropriate for directly linking their measurements to wood anatomy. New and upgraded needle head shapes should first be explored in conifers, since their wood structure, in terms of different cell types, is less complex than that of broadleaves due to being evolutionarily older and the interpretation of potential relationships is therefore simpler.

Since wood density in conifers mainly depends on the average size and amount of wall material fixed in the tracheids (Björklund et al., 2017), the relationships between resistance drilling density and wood-anatomical features in conifers should be relatively straightforward (i.e., if the frequency of IADSs is minimal)—assuming that the shape of the needle head is adjusted to minimize interferences from the drilling process and to maximize measurement sensitivity. Different lengths over which resistance drilling density and anatomical features are being linked should also be explored in future research, to reduce the impact of localized changes in TRW and, consequently, the amount of wall material fixed in tracheids, on these relationships. In addition, TRW of analyzed trees should be comparable or at least accounted for in future studies.

## Conclusion

In our study, the relationships between tree-ring width, wood anatomy, and resistance drilling density were demonstrated by using material-centered approach. The increment width and wood anatomy data were presented only for the first 7 cm of the collected cores because it was not possible to synchronize wood anatomy data and resistance drilling density at annual levels. In general, the relationships between increment widths and wood-anatomical features show similar results when compared to studies conducted at the annual level. Resistance drilling densities showed weak correlations with anatomical features in spruce and no correlations in beech. Resistance drilling density measurements have been shown to be a rapid and reliable means of assessing differences in wood density between species or even within species (Krajnc et al., 2022). We propose to improve resistance drilling density measurements to increase resolution and accuracy so that we can assess variation at the intra-annual level. In addition, the methodology should be compared with other established methods, such as *x*-ray density measurements. If possible, different *x*-ray devices should be used to also assess potential differences in density measurements between devices. Furthermore, the quantitative wood anatomy measurements and analysis should also be synchronized; namely, in the case of beech, only conducting cells were analyzed, while in the case of spruce, all tracheids were considered in the analysis. Such studies would improve our understanding of how environmental conditions affect tree ring and wood-anatomical features, as well as density as the main wood quality indicator.

## Data Availability Statement

The raw data supporting the conclusions of this article will be made available by the authors, without undue reservation.

## Author Contributions

PP, JG, LK, and DA planned and designed the research and wrote the manuscript. DA, PP, and LK performed the sampling. DA contributed to the sample preparation and capturing of high-resolution images with light microscope and analysis with Roxas and Image-Pro Plus. DA and LK performed and analyzed the resistograph drilling measurements. All authors contributed to the article and approved the submitted version.

## Funding

This work was supported by the Slovenian Research Agency, young researchers’ program (DA), program P4-0107 and P4-0430, and projects Z4-7318 and J4-2541.

## Conflict of Interest

The authors declare that the research was conducted in the absence of any commercial or financial relationships that could be construed as a potential conflict of interest.

## Publisher’s Note

All claims expressed in this article are solely those of the authors and do not necessarily represent those of their affiliated organizations, or those of the publisher, the editors and the reviewers. Any product that may be evaluated in this article, or claim that may be made by its manufacturer, is not guaranteed or endorsed by the publisher.
