# Supplementary material for: Relationships Between Wood-Anatomical Features and Resistance Drilling Density in Norway Spruce and European Beech
Source: Front Plant Sci. 2022 Apr 8;13:872950. doi: 10.3389/fpls.2022.872950 (PMC9024210; doi:10.3389/fpls.2022.872950)
Supplement: Supplementary file 1 [file Data_Sheet_1.docx]

Supplementary Material

# Supplementary Figures and Tables

## Supplementary Figures


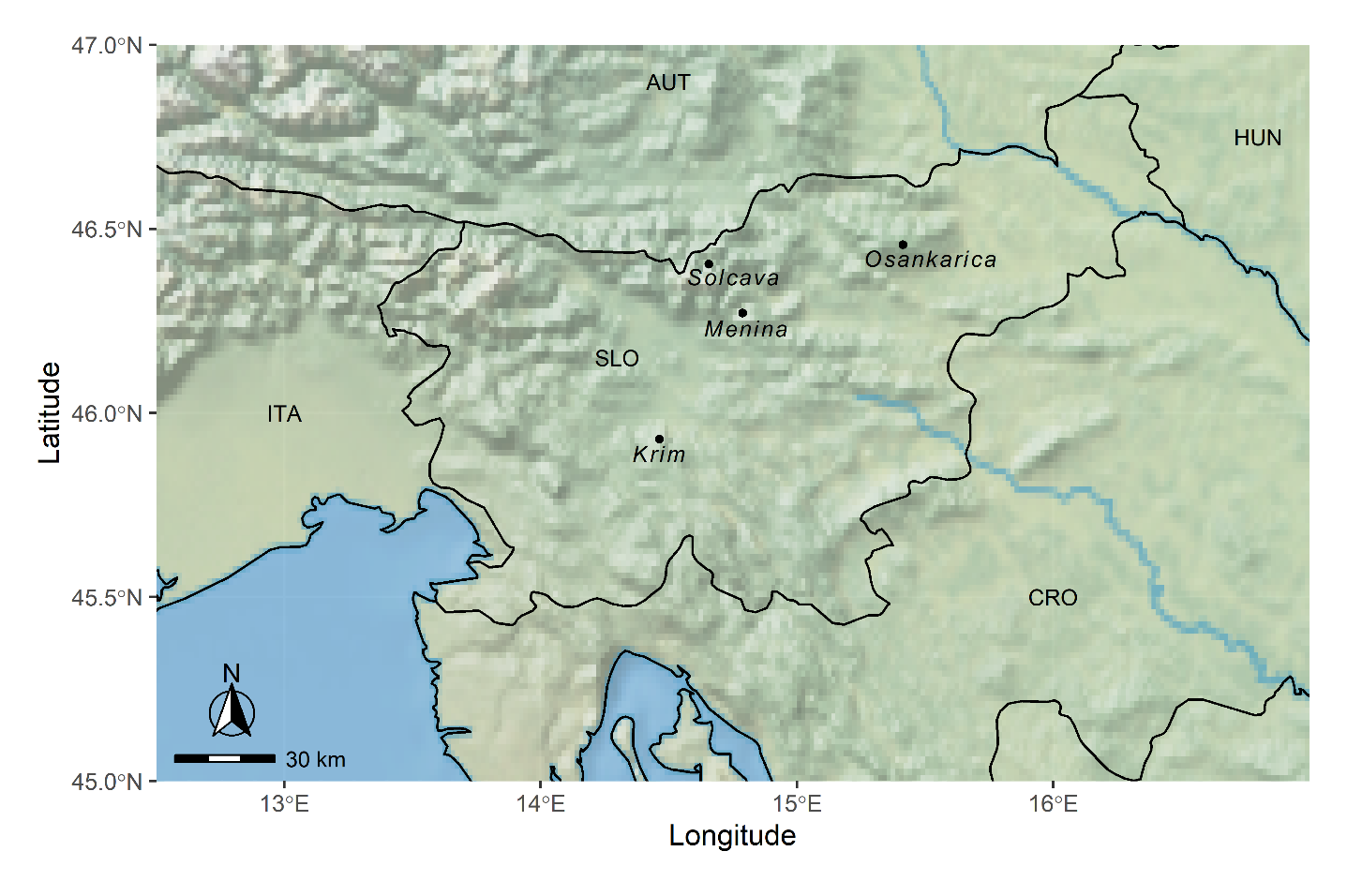


**Supplementary Figure 1.** Geographical locations of selected sites (Solcava, Menina, Krim and Osankarica).


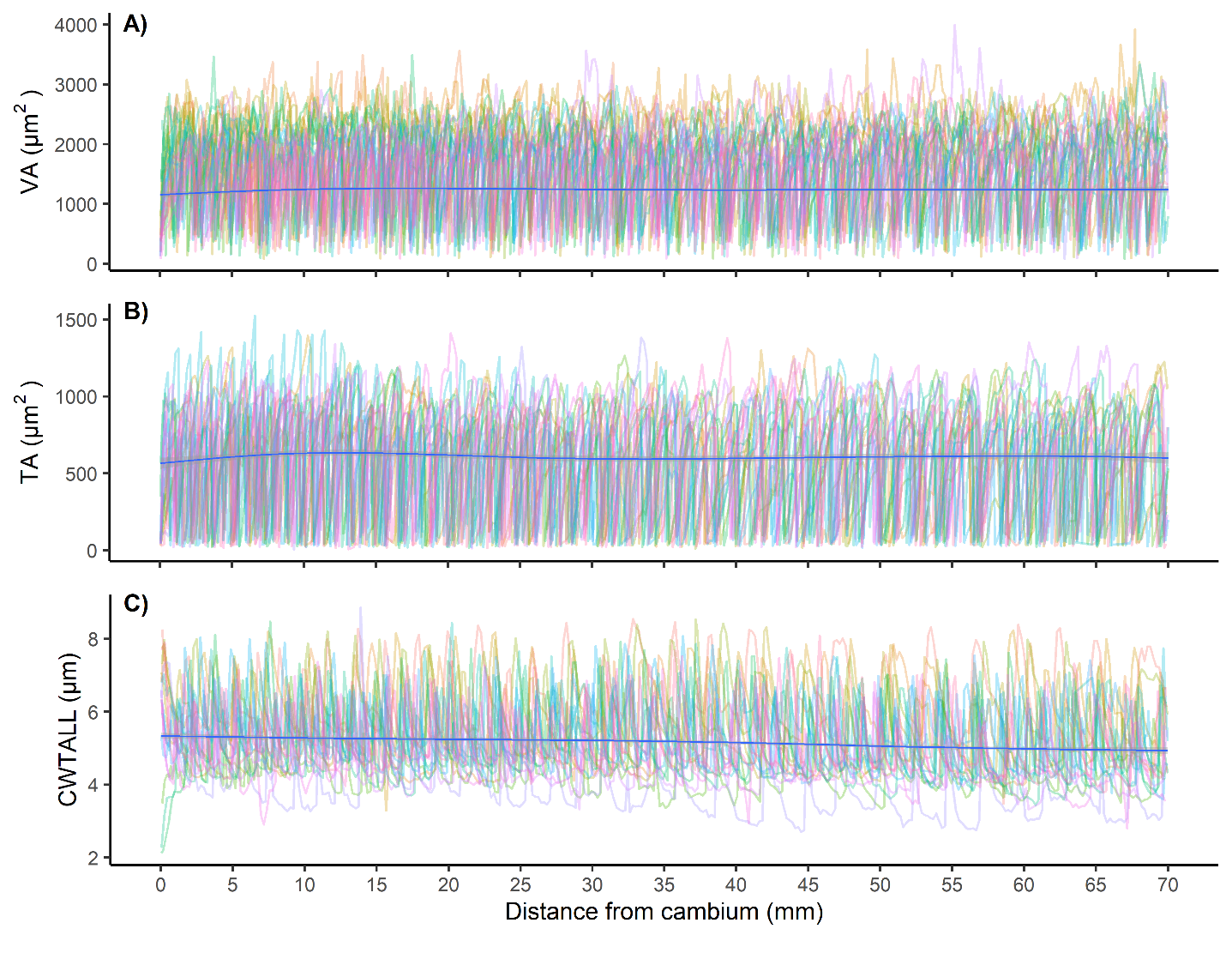


**Supplementary Figure 2.** Raw measured data of wood anatomical features (beech vessel area – VA, spruce tracheid area – TA and spruce tracheid mean cell wall thickness – CWTALL) in examined 7 cm of selected wood cores. Blue lines representing mean of selected wood anatomical features, while background lines representing the raw variability of analyzed data.

1. **Supplementary Tables**

**Supplementary Table 1:** Additional information about tree (diameter at breast height – DBH (cm), tree height – Height (m) and age) and site characteristics (Altitude (m) mean annual temperature – MAT(°C) and mean annual precipitation sum – MAP(mm)) for four selected sites in Slovenia.

| Site | Species | Tree characteristics | | | Site characteristics | | |
| --- | --- | --- | --- | --- | --- | --- | --- |
|  |  | DBH (cm) | Height (m) | Age (years) | Altitude (m) | MAT (°C) | MAP (mm) |
| Menina | Norway spruce | 63.8 | 32.6 | 131.0 | 1100.0 | 7.2 | 1512.0 |
|  | European beech | 60.6 | 31.4 | 136.0 |  |  |  |
| Solcava | Norway spruce | 49.3 | 28.9 | 110.0 | 1320.0 | 5.9 | 1488.0 |
|  | European beech | 49.5 | 29.6 | 140.0 |  |  |  |
| Krim | Norway spruce | 51.6 | 33.4 | 90.0 | 960.0 | 9.2 | 1462.0 |
|  | European beech | 51.4 | 33.2 | 117.0 |  |  |  |
| Osankarica | Norway spruce | 45.5 | 29.7 | 114.0 | 1240.0 | 6.3 | 1190.0 |
|  | European beech | 47.2 | 29.0 | 120.0 |  |  |  |
| ALL - mean | Norway spruce | 52.6 | 31.2 | 111.3 | 1155.0 | 7.2 | 1413.0 |
|  | European beech | 52.2 | 30.8 | 128.3 |  |  |  |
| ALL - st. dev. | Norway spruce | 7.9 | 2.2 | 16.8 | 158.6 | 1.5 | 150.1 |
|  | European beech | 5.9 | 1.9 | 11.4 |  |  |  |

**Supplementary Table 2:**Pearson correlation coefficients between wood anatomy features (TRW – tree ring widths, CD – cell density, RCTA – relative conduit area, MTA – mean tracheid area, DRAD – radial tracheid diameter, DTAN – tangential tracheid diameter, CWTALL – mean tracheid cell wall thickness, CWTRAD - mean radial tracheid cell wall thickness, CWTTAN - mean tangential tracheid cell wall thickness, LWS – Late wood share in TRW, TWS+LWS – sum of transitional- and late wood share in TRW, LWW – Late wood width and TWW+LWW sum of transitional- and late wood width) and wood density quartiles (from fifth wood density quantile – WD-q5 till 95^th^ quantile – WD-q95) in spruce. Bold and grey colored values representing significant correlations, where p<0.05 significance level was considered.

|  | TRW | CD | RCTA | MTA | DRAD | DTAN | CWTALL | CWTRAD | CWTTAN | LWS | TWS+ LWS | LWW | TWW+ LWW | WD-q5 | WD-q10 | WD-q25 | WD-q50 | WD-q75 | WD-q90 | WD-q95 |
| --- | --- | --- | --- | --- | --- | --- | --- | --- | --- | --- | --- | --- | --- | --- | --- | --- | --- | --- | --- | --- |
| TRW | 1 | -0.19 | -0.15 | 0.01 | 0.03 | 0.02 | 0.26 | 0.29 | 0.22 | 0.33 | 0.25 | **0.84** | **0.84** | 0.04 | 0.02 | 0.02 | 0.17 | 0.32 | 0.34 | 0.35 |
| CD | -0.19 | 1 | -0.3 | **-0.74** | **-0.67** | **-0.75** | 0.02 | 0.02 | 0 | 0.47 | 0.16 | 0.11 | -0.06 | 0.23 | 0.24 | 0.25 | 0.26 | 0.27 | 0.24 | 0.22 |
| RCTA | -0.15 | -0.3 | 1 | **0.85** | **0.88** | **0.69** | **-0.94** | **-0.93** | **-0.94** | **-0.89** | **-0.95** | **-0.59** | **-0.63** | -0.13 | -0.15 | -0.25 | -0.34 | -0.42 | -0.41 | -0.41 |
| MTA | 0.01 | **-0.74** | **0.85** | 1 | **0.96** | **0.91** | **-0.66** | **-0.65** | **-0.65** | **-0.87** | **-0.75** | -0.47 | -0.4 | -0.28 | -0.3 | -0.37 | -0.43 | -0.48 | -0.46 | -0.45 |
| DRAD | 0.03 | **-0.67** | **0.88** | **0.96** | 1 | **0.78** | **-0.71** | **-0.7** | **-0.71** | **-0.85** | **-0.75** | -0.45 | -0.4 | -0.08 | -0.1 | -0.18 | -0.24 | -0.3 | -0.27 | -0.26 |
| DTAN | 0.02 | **-0.75** | **0.69** | **0.91** | **0.78** | 1 | **-0.49** | **-0.49** | -0.47 | **-0.82** | **-0.64** | -0.42 | -0.31 | -0.45 | -0.47 | **-0.53** | **-0.58** | **-0.61** | **-0.62** | **-0.62** |
| CWTALL | 0.26 | 0.02 | **-0.94** | **-0.66** | **-0.71** | **-0.49** | 1 | **1** | **1** | **0.8** | **0.92** | **0.62** | **0.7** | 0.09 | 0.12 | 0.21 | 0.29 | 0.37 | 0.35 | 0.34 |
| CWTRAD | 0.29 | 0.02 | **-0.93** | **-0.65** | **-0.7** | **-0.49** | **1** | 1 | **0.99** | **0.81** | **0.91** | **0.64** | **0.72** | 0.11 | 0.13 | 0.22 | 0.3 | 0.38 | 0.36 | 0.36 |
| CWTTAN | 0.22 | 0 | **-0.94** | **-0.65** | **-0.71** | -0.47 | **1** | **0.99** | 1 | **0.78** | **0.92** | **0.59** | **0.68** | 0.07 | 0.1 | 0.19 | 0.27 | 0.34 | 0.32 | 0.32 |
| LWS | 0.33 | 0.47 | **-0.89** | **-0.87** | **-0.85** | **-0.82** | **0.8** | **0.81** | **0.78** | 1 | **0.86** | **0.77** | **0.7** | 0.14 | 0.16 | 0.24 | 0.36 | 0.46 | 0.47 | 0.48 |
| TWS+LWS | 0.25 | 0.16 | **-0.95** | **-0.75** | **-0.75** | **-0.64** | **0.92** | **0.91** | **0.92** | **0.86** | 1 | **0.66** | **0.73** | 0.21 | 0.23 | 0.33 | 0.41 | **0.49** | **0.49** | **0.5** |
| LWW | **0.84** | 0.11 | **-0.59** | -0.47 | -0.45 | -0.42 | **0.62** | **0.64** | **0.59** | **0.77** | **0.66** | 1 | **0.96** | 0.11 | 0.11 | 0.15 | 0.31 | 0.46 | 0.48 | **0.48** |
| TWW+LWW | **0.84** | -0.06 | **-0.63** | -0.4 | -0.4 | -0.31 | **0.7** | **0.72** | **0.68** | **0.7** | **0.73** | **0.96** | 1 | 0.13 | 0.13 | 0.19 | 0.33 | 0.48 | **0.49** | **0.49** |
| WD-q5 | 0.04 | 0.23 | -0.13 | -0.28 | -0.08 | -0.45 | 0.09 | 0.11 | 0.07 | 0.14 | 0.21 | 0.11 | 0.13 | 1 | **1** | **0.99** | **0.96** | **0.89** | **0.84** | **0.81** |
| WD-q10 | 0.02 | 0.24 | -0.15 | -0.3 | -0.1 | -0.47 | 0.12 | 0.13 | 0.1 | 0.16 | 0.23 | 0.11 | 0.13 | **1** | 1 | **0.99** | **0.96** | **0.89** | **0.85** | **0.81** |
| WD-q25 | 0.02 | 0.25 | -0.25 | -0.37 | -0.18 | **-0.53** | 0.21 | 0.22 | 0.19 | 0.24 | 0.33 | 0.15 | 0.19 | **0.99** | **0.99** | 1 | **0.98** | **0.91** | **0.87** | **0.84** |
| WD-q50 | 0.17 | 0.26 | -0.34 | -0.43 | -0.24 | **-0.58** | 0.29 | 0.3 | 0.27 | 0.36 | 0.41 | 0.31 | 0.33 | **0.96** | **0.96** | **0.98** | 1 | **0.98** | **0.95** | **0.93** |
| WD-q75 | 0.32 | 0.27 | -0.42 | -0.48 | -0.3 | **-0.61** | 0.37 | 0.38 | 0.34 | 0.46 | **0.49** | 0.46 | **0.48** | **0.89** | **0.89** | **0.91** | **0.98** | 1 | **0.99** | **0.97** |
| WD-q90 | 0.34 | 0.24 | -0.41 | -0.46 | -0.27 | **-0.62** | 0.35 | 0.36 | 0.32 | 0.47 | **0.49** | 0.48 | **0.49** | **0.84** | **0.85** | **0.87** | **0.95** | **0.99** | 1 | **0.99** |
| WD-q95 | 0.35 | 0.22 | -0.41 | -0.45 | -0.26 | **-0.62** | 0.34 | 0.36 | 0.32 | 0.48 | **0.5** | **0.48** | **0.49** | **0.81** | **0.81** | **0.84** | **0.93** | **0.97** | **0.99** | 1 |

**Supplementary Table 3**: Pearson correlation coefficients between wood anatomy features (TRW – tree ring widths, CD – cell density, RCTA – relative conduit area, MVA – mean vessel area, DRAD – radial vessel diameter, DTAN – tangential vessel diameter) and wood density quartiles (from fifth wood density quantile – WD-q5 till 95^th^ quantile – WD-q95) in beech. Bold and grey colored values representing significant correlations, where p<0.05 significance level was considered.

|  | TRW | CD | RCTA | MVA | DRAD | DTAN | WD-q5 | WD-q10 | WD-q25 | WD-q50 | WD-q75 | WD-q90 | WD-q95 |
| --- | --- | --- | --- | --- | --- | --- | --- | --- | --- | --- | --- | --- | --- |
| TRW | 1 | **-0.55** | -0.28 | 0.12 | 0.34 | 0 | 0.33 | 0.31 | 0.29 | 0.3 | 0.3 | 0.31 | 0.3 |
| CD | **-0.55** | 1 | **0.74** | 0.09 | -0.07 | 0.17 | -0.33 | -0.32 | -0.3 | -0.29 | -0.29 | -0.28 | -0.26 |
| RCTA | -0.28 | **0.74** | 1 | **0.73** | **0.52** | **0.75** | -0.26 | -0.26 | -0.25 | -0.24 | -0.24 | -0.21 | -0.19 |
| MVA | 0.12 | 0.09 | **0.73** | 1 | **0.84** | **0.96** | -0.12 | -0.14 | -0.15 | -0.14 | -0.14 | -0.11 | -0.09 |
| DRAD | 0.34 | -0.07 | **0.52** | **0.84** | 1 | **0.68** | -0.01 | -0.01 | 0 | 0.02 | 0.04 | 0.07 | 0.09 |
| DTAN | 0 | 0.17 | **0.75** | **0.96** | **0.68** | 1 | -0.19 | -0.21 | -0.23 | -0.22 | -0.23 | -0.2 | -0.18 |
| WD-q5 | 0.33 | -0.33 | -0.26 | -0.12 | -0.01 | -0.19 | 1 | **1** | **0.99** | **0.98** | **0.97** | **0.97** | **0.96** |
| WD-q10 | 0.31 | -0.32 | -0.26 | -0.14 | -0.01 | -0.21 | **1** | 1 | **1** | **0.99** | **0.98** | **0.98** | **0.97** |
| WD-q25 | 0.29 | -0.3 | -0.25 | -0.15 | 0 | -0.23 | **0.99** | **1** | 1 | **1** | **0.99** | **0.99** | **0.98** |
| WD-q50 | 0.3 | -0.29 | -0.24 | -0.14 | 0.02 | -0.22 | **0.98** | **0.99** | **1** | 1 | **1** | **0.99** | **0.99** |
| WD-q75 | 0.3 | -0.29 | -0.24 | -0.14 | 0.04 | -0.23 | **0.97** | **0.98** | **0.99** | **1** | 1 | **1** | **0.99** |
| WD-q90 | 0.31 | -0.28 | -0.21 | -0.11 | 0.07 | -0.2 | **0.97** | **0.98** | **0.99** | **0.99** | **1** | 1 | **1** |
| WD-q95 | 0.3 | -0.26 | -0.19 | -0.09 | 0.09 | -0.18 | **0.96** | **0.97** | **0.98** | **0.99** | **0.99** | **1** | 1 |

**Supplementary Table 4**: Supplementary information for linear models between spruce mean resistance drilling density (RDD) and wood anatomical features (MTA – mean tracheid area, RCTA – relative conduit area, DRAD – radial tracheid diameter, DTAN – tangential tracheid diameter, TRW – tree ring widths, CWTALL – mean tracheid cell wall thickness, LWW – Late wood width and TWW+LWW - sum of transitional- and late wood width) in Figure 6.

| lm(formula = RDD ~ MTA) | | | | R^2^ =0.18 | | |
| --- | --- | --- | --- | --- | --- | --- |
|  | Estimate | Std Error | T value | | Pr(>\|t\|) | siq |
| Intercept | 400.7 | 44.1 | 9.1 | | <0.0001 | *** |
| MTA | -0.15 | 0.08 | -1.9 | | 0.08 | . |
| lm(formula = RDD ~ RCTA) | | | |  | | |
|  | Estimate | Std Error | T value | | Pr(>\|t\|) | siq |
| Intercept | 409.3 | 64.9 | 6.3 | | <0.0001 | *** |
| RCTA | -1.85 | 1.3 | -1.4 | | 0.19 | ns |
| lm(formula = RDD ~ DRAD) | | | |  | | |
|  | Estimate | Std Error | T value | | Pr(>\|t\|) | siq |
| Intercept | 380.8 | 64.1 | 5.9 | | <0.0001 | *** |
| DRAD | -2.4 | 2.5 | -0.9 | | 0.36 | ns |
| lm(formula = RDD ~ DTAN) | | | | R^2^ =0.34 | | |
|  | Estimate | Std Error | T value | | Pr(>\|t\|) | siq |
| Intercept | 552.9 | 84.4 | 6.5 | | <0.0001 | *** |
| DTAN | -9.8 | 3.5 | -2.8 | | 0.014 | * |
| lm(formula = RDD ~ TRW) | | | |  | | |
|  | Estimate | Std Error | T value | | Pr(>\|t\|) | siq |
| Intercept | 306 | 22.05 | 13.9 | | <0.0001 | *** |
| TRW | 0.007 | 0.01 | 0.66 | | 0.51 | ns |
| lm(formula = RDD ~ CWTALL) | | | |  | | |
|  | Estimate | Std Error | T value | | Pr(>\|t\|) | siq |
| Intercept | 244.9 | 65.4 | 3.7 | | 0.002 | ** |
| CWTALL | 14.12 | 12.2 | 1.2 | | 0.26 | ns |
| lm(formula = RDD ~ LWW) | | | |  | | |
|  | Estimate | Std Error | T value | | Pr(>\|t\|) | siq |
| Intercept | 305.1 | 13.8 | 22.15 | | <0.0001 | *** |
| LWW | 0.05 | 0.04 | 1.3 | | 0.22 | ns |
| lm(formula = RDD ~ LWW+ TWW) | | | |  | | |
|  | Estimate | Std Error | T value | | Pr(>\|t\|) | siq |
| Intercept | 302.2 | 14.7 | 20.6 | | <0.0001 | *** |
| LWW+TWW | 0.02 | 0.02 | 1.4 | | 0.19 | ns |

**Supplementary Table 5**: Supplementary information for linear models between spruce 90^th^ quantile of resistance drilling density (RDDq90) and wood anatomical features (MTA – mean tracheid area, RCTA – relative conduit area, DRAD – radial tracheid diameter, DTAN – tangential tracheid diameter, TRW – tree ring widths, CWTALL – mean tracheid cell wall thickness, LWW – Late wood width and TWW+LWW - sum of transitional- and late wood width) in Figure 7.

| lm(formula = RDDq90 ~ MTA) | | | | R^2^ =0.21 | | |
| --- | --- | --- | --- | --- | --- | --- |
|  | Estimate | Std Error | T value | | Pr(>\|t\|) | siq |
| Intercept | 441.5 | 42.5 | 10.4 | | <0.0001 | *** |
| MTA | -0.15 | 0.08 | -2.0 | | 0.06 | . |
| lm(formula = RDDq90 ~ RCTA) | | | | R^2^ =0.17 | | |
|  | Estimate | Std Error | T value | | Pr(>\|t\|) | siq |
| Intercept | 463.1 | 61.5 | 7.5 | | <0.0001 | *** |
| RCTA | -2.2 | 1.3 | -1.7 | | 0.10 | . |
| lm(formula = RDDq90~ DRAD) | | | |  | | |
|  | Estimate | Std Error | T value | | Pr(>\|t\|) | siq |
| Intercept | 425.4 | 62.1 | 6.8 | | <0.0001 | *** |
| DRAD | -2.6 | 2.4 | -1.1 | | 0.29 | ns |
| lm(formula = RDDq90~ DTAN) | | | | R^2^ =0.38 | | |
|  | Estimate | Std Error | T value | | Pr(>\|t\|) | siq |
| Intercept | 599.1 | 79.9 | 7.5 | | <0.0001 | *** |
| DTAN | -10.2 | 3.4 | -3.0 | | 0.008 | ** |
| lm(formula = RDDq90~ TRW) | | | |  | | |
|  | Estimate | Std Error | T value | | Pr(>\|t\|) | siq |
| Intercept | 330 | 20.6 | 16.0 | | <0.0001 | *** |
| TRW | 0.01 | 0.01 | 1.4 | | 0.18 | ns |
| lm(formula = RDDq90~ CWTALL) | | | |  | | |
|  | Estimate | Std Error | T value | | Pr(>\|t\|) | siq |
| Intercept | 268.7 | 62.6 | 4.3 | | <0.0001 | *** |
| CWTALL | 16.7 | 11.7 | 1.4 | | 0.17 | ns |
| lm(formula = RDDq90~ LWW) | | | | R^2^ =0.23 | | |
|  | Estimate | Std Error | T value | | Pr(>\|t\|) | siq |
| Intercept | 334.7 | 12.5 | 26.9 | | <0.0001 | *** |
| LWW | 0.08 | 0.04 | 2.11 | | 0.05 | . |
| lm(formula = RDDq90~ LWW+TWW) | | | | R^2^ =0.24 | | |
|  | Estimate | Std Error | T value | | Pr(>\|t\|) | siq |
| Intercept | 332.1 | 13.3 | 24.9 | | <0.0001 | *** |
| LWW+ TWW | 0.04 | 0.02 | 2.2 | | 0.04 | * |

**Supplementary Table 6**: Supplementary information for linear models between beech mean resistance drilling density (RDD) and wood anatomical features (MVA – mean vessel area, RCTA – relative conduit area, DRAD – radial vessel diameter, DTAN – tangential vessel diameter, TRW – tree ring widths) in Figure 8.

| lm(formula = RDD ~ MVA) | | | |  | | |
| --- | --- | --- | --- | --- | --- | --- |
|  | Estimate | Std Error | T value | | Pr(>\|t\|) | siq |
| Intercept | 495.5 | 82.2 | 6.03 | | <0.0001 | *** |
| MVA | -0.03 | 0.05 | -0.64 | | 0.52 | ns |
| lm(formula = RDD ~ RCTA) | | | |  | | |
|  | Estimate | Std Error | T value | | Pr(>\|t\|) | siq |
| Intercept | 504.7 | 53.8 | 9.4 | | <0.0001 | *** |
| RCTA | -3.05 | 2.6 | -1.16 | | 0.26 | ns |
| lm(formula = RDD ~ DRAD) | | | |  | | |
|  | Estimate | Std Error | T value | | Pr(>\|t\|) | siq |
| Intercept | 422.1 | 183.9 | 2.3 | | 0.03 | * |
| DRAD | 0.42 | 3.7 | -0.11 | | 0.91 | ns |
| lm(formula = RDD ~ DTAN) | | | |  | | |
|  | Estimate | Std Error | T value | | Pr(>\|t\|) | siq |
| Intercept | 592.1 | 140.1 | 4.3 | | <0.0001 | *** |
| DTAN | -3.7 | 3.5 | -1.07 | | 0.30 | ns |
| lm(formula = RDD ~ TRW) | | | |  | | |
|  | Estimate | Std Error | T value | | Pr(>\|t\|) | siq |
| Intercept | 395.9 | 33.3 | 11.9 | | <0.0001 | *** |
| TRW | 0.03 | 0.02 | 1.5 | | 0.16 | ns |
